# Anisotropic Resistive Switching in NiO Thin Films Deposited on Stepped MgO Substrates

**DOI:** 10.3390/nano15221703

**Published:** 2025-11-11

**Authors:** Tolagay Duisebayev, Mergen Zhazitov, Muhammad Abdullah, Yerbolat Tezekbay, Askar Syrlybekov, Margulan Ibraimov, Bakyt Khaniyev, Timur Serikov, Nurxat Nuraje, Olzat Toktarbaiuly

**Affiliations:** 1 National Laboratory Astana, Nazarbayev University, Astana 010000, Kazakhstan; tolagay.duisebayev@nu.edu.kz (T.D.);; 2 Department of Physics and Technology, Al-Farabi Kazakh National University, Almaty 050040, Kazakhstankhaniyev.bakyt@gmail.com (B.K.); 3 Institute of Molecular Nanophotonics, Buketov Karaganda University, Karaganda 100024, Kazakhstan; 4 Department of Chemical & Materials Engineering, School of Engineering & Digital Science, Nazarbayev University, Astana 010000, Kazakhstan; nurxat.nuraje@nu.edu.kz; 5 TEQNOVATE LLC, Astana 010000, Kazakhstan

**Keywords:** resistive random-access memory, nickel oxide, anisotropic switching, stepped MgO substrate

## Abstract

Thin films of nickel oxide (NiO) were deposited on a 5° miscut magnesium oxide (MgO)(100) substrate using electron-beam evaporation to pursue morphology-directed resistive switching. The atomic force microscope (AFM) confirmed a stepped surface with a terrace width of ~85 nm and a step height of ~7 nm. After deposition, the film resistance decreased from 200 MΩ to 25 MΩ by annealing under ambient air at 400 °C, attributed to the increase in the p-type conductivity through nickel vacancy formation. Top electrodes of Ag (500 nm width, 180 nm gap) were patterned parallel or perpendicular to the substrate steps using UV and electron-beam lithography. Devices aligned parallel to the step showed reproducible unipolar switching with 100% yield between forming voltages 20–70 V and HRS/LRS~10^2^ at ±5 V. In contrast, devices formed perpendicular to the steps (8/8) subsequently failed catastrophically during electroforming, with scanning electron microscopy (SEM) showing breakdown holes on the order of ~100 nm at the step crossings. The anisotropic electrodynamic response is due to step-guided electric field distribution and directional nickel vacancy migration, illustrating how substrate morphology can deterministically influence filament nucleation. These results highlighted stepped MgO as a template to engineer the anisotropic charge transport of NiO, exhibiting a reliable ReRAM as well as directional electrocatalysis for energy applications.

## 1. Introduction

Thin-film resistive switching has become one of the most promising candidates for next-generation, non-volatile memory devices, or resistive random-access memory (ReRAM) [1,2,3,4,5]. This capability stems from its ability to mitigate the drawbacks of conventional charge memories by presenting improved scalability, increased switching speed, lower amounts of power consumption, and improved endurance. Although an industrial ReRAM deployment mandates extreme levels of endurance and retention, basic research that addresses issues associated with switching reproducibility and yield, which are two considerable obstacles of filamentary devices, is also what is fundamentally needed for scalable integration. The basic resistive switching mechanism involves reversible modification of a material’s state of resistance as a function of its applied electric field. Change is primarily governed by defect mobility, especially by the migration of oxygen vacancies, cation redistribution, and filamentary conduction pathways through oxide films [6]. Of the many materials that have been studied for resistive switching, of great interest has been NiO. NiO is a simple binary transition metal oxide in a rock-salt structure, found readily available, has suitable environmental stability, and is relatively easy to fabricate. Most importantly for this context is that NiO has correctly defined resistive switching behavior that can be regulated by the growth conditions, defect manipulation, and electrical contact geometries [7,8].

NiO’s origins of resistive switching are connected closely to its high density of intrinsic defects (e.g., nickel and oxygen vacancies) that exert strict control of local electronic transport. Accordingly, NiO presents both an ideal model system to understand defect-induced switching mechanisms and a realistic candidate for incorporation into devices in the future. However, improvements in performance and reliability of NiO-based resistive switching devices necessitate the control of film quality, crystallographic orientation, and defect density. An effective means of achieving this is with substrate engineering. MgO is a common substrate material for NiO thin films owing to a close structural compatibility: they both have a rock-salt structure and similar lattice parameters that allow for epitaxy with high film quality [9,10,11,12,13]. Additionally, MgO substrates potentially have unique utility, avoiding lattice matching, if grown with a vicinal miscut leading to a stepped surface structure defined by terraces and atomic steps. Stepped substrates provide controlled anisotropy, local strain fields, and preferential nucleation areas that can provide significant control overgrowth, film structure, and defect densities in top NiO films. The interplay of energy involved in step formation, terrace size, and adatom mobility in vicinal systems establishes which growth mode, step-flow, layer-by-layer, or islanding will dominate, and all these growth modes will significantly impact defect distribution and transport anisotropy [13,14,15,16,17].

More recently, it has also been shown that anisotropy due to the substrate controls the resistive switching: Tezekbay et al. showed that Fe_3_O_4_ films grown on stepped MgO substrates exhibited anisotropic resistive switching, in which the electric field orientation strongly modified both switching voltage and performance as a function of temperature [18]. Tezekbay et al. also took advantage of a 5° miscut MgO substrate to obtain well-defined step arrays in Fe_3_O_4_, establishing this angle as a standard for anisotropic studies of rock-salt oxides. Thus, while this study establishes stepped substrates as a means of controlling switching anisotropy, the underlying mechanisms are obscured by the mixed-valence and magnetic complexity of Fe_3_O_4_. As a second case, the system of NiO—a simple rock-salt p-type oxide driven primarily by nickel vacancy motion—provides a cleaner example to disentangle the role of geometric anisotropy in defect-mediated transport. In this context, we show that step orientation minimizes forming voltages while simultaneously determining anisotropic filament nucleation pathways, therefore allowing reproducible switching only when the applied electric field is parallel to the substrate step edge. This work demonstrates a universal concept that substrate steps will act as directional templates for ionic migration, for any defect-driven oxide system with epitaxial compatibility and anisotropic surface morphology. The presence of steps on MgO surfaces adds yet an additional tunability dimension to thin-film growth kinetics. Step edges provide energetically favorable environments for the incorporation of adatoms, potentially producing anisotropic defect distributions or vacancy generation. Stepped surfaces can also impact domain alignment, give rise to surface roughness, and add localized strain relaxation, all of which are critical effects for electronic transport in NiO [19,20,21,22,23,24].

Earlier studies have demonstrated that these can have a strong influence on functional properties of epitaxial oxides; thus, substrate morphology is now an important consideration in device fabrication [25,26,27,28,29,30]. For resistive switching, where filament growth and charge migration are highly susceptible to defects, substrate-induced effects can be a very powerful modulator of device performance. It should be noted that NiO is a multifunctional material which extends well beyond memories. As a p-type wide-bandgap semiconductor, NiO has been investigated in photovoltaics, transparent electrodes, and other electrochemical devices. For example, NiO-based oxide heterostructures have been studied for UV photovoltaics due to their transparency and rectifying behavior [31].

In electrochemistry, NiO is known to function more like a pure catalyst (or non-precious electrocatalyst), utilizing, for example, its dimensions, morphology, and/or defect density to facilitate the oxygen evolution reaction (OER) reaction [32,33]. Although this work addresses resistive switching, the substrate-mediated anisotropy described is also suitable for photoelectrochemical water splitting. Specifically, establishing the substrate-modulated charge migration pathways and defect distributions may offer a viable strategy for boosting the performance of NiO on a range of functional platforms. To examine anisotropy, we create Ag top electrodes (width of 500 nm, gap of 180 nm) and use either UV or electron-beam (e-beam) lithography to position them parallel or normal to the substrate steps—designed to decouple the effect of step orientation on filament formation. Here, we examine the stepped MgO substrate effects on NiO thin film resistive switching properties. A vicinal miscut MgO substrate (5° miscut) was subjected to thermal annealing to create a well-defined step morphology as observed by AFM. NiO thin films were subsequently deposited by e-beam evaporation, and post deposition annealing was used to affect nickel vacancy concentration and to establish the p-type semiconducting characteristics of the thin films. Despite the fact that the stepped single-crystal MgO substrate calls for high-temperature (≥1200 °C) annealing, resulting in a lack of compatibility with CMOS integration, it is possible to leverage the design principle of steps in anisotropic, step-directed defect migration paths on Si-based platforms with nanostructured templates or engineered buffer layers that allow for low-temperature (<400 °C) processing and can be incorporated into back-end-of-line (BEOL) processing.

The films’ morphology and structure were examined in detail, while the electrical properties were studied in parallel and perpendicular fields to examine any anisotropic effect. Device development included using UV and e-beam lithography to define the electrode pattern with high fidelity, thereby allowing for current–voltage (I–V) measurements of the resistive switching behavior. An electrical performance exhibited extreme anisotropy due to the step orientation, which was an essential factor in determining the switching behavior and established voltage. These studies highlighted the importance of substrate-induced anisotropy in defect-switching processes and offered a new angle on using substrate morphology to take advantage in oxide-based memory devices. Concurrently, the results suggested universal design rules for catalytic and energy conversion systems, where directional charge transport and defect engineering also emerge as a design principle.

## 2. Experimental Process

NiO thin films were deposited on stepped MgO substrates using an Edwards electron-beam evaporation system. Prior to characterization, the films underwent post-annealing in an ambient air environment to enhance their structural and electrical properties. Immediately following the deposition process, AFM and SEM techniques were used to characterize NiO films. Contacts were designed and manufactured using ultraviolet (UV) and electron-beam lithography (EBL) techniques, suitable for measuring electrical properties using a probe station by utilizing current–voltage (I–V) measurement.

**Figure 1:**
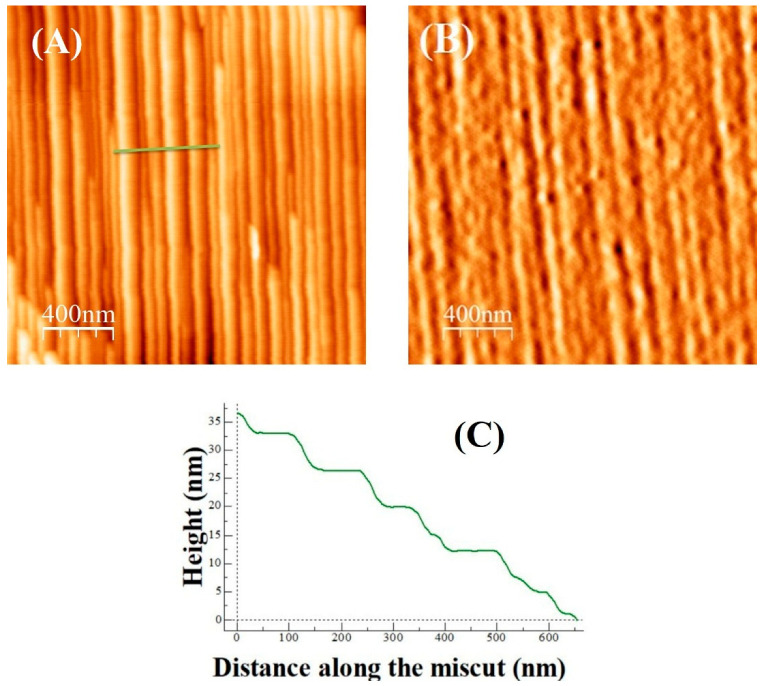
Characterization of the MgO(100) substrate with a 5° miscut using AFM. (**A**) Topographic image showing well-formed terraces and steps aligned in one direction. The green line indicates the height profile shown below. (**B**) An image of one of the terraces taken at higher magnification, showing that the surfaces are relatively smooth and with low defect density. (**C**) A line profile in the miscut face direction confirming the average step height of ~7 nm and the average terrace width of ~85 nm consistent with the 5° miscut reported.

The substrates used for thin-film deposition are vicinal 5° miscut MgO(100) templates. The miscut angle is selected to provide a compromise between sufficient step density for anisotropic templating and the continuity of the film: it provides regular terraces of ~85 nm width and ~7 nm step height dimensions known to facilitate epitaxial growth and defect migration without inducing step bunching. Small miscuts (<2°) produce too little step density for any measurable anisotropy; larger miscuts (>7°) frequently lead to morphologic instability. The 5° configuration is the establishment in oxide heteroepitaxy to probe step-directed phenomena [18,21]. Before deposition on good thin films, the MgO substrates were annealed at 1200 °C for 3 h in air. This annealing treatment facilitates the development of stepped surface structures characterized by well-defined terraces. The morphology of the annealed substrates was assessed using AFM imaging. Figure 1A displays a topographic scan showing distinct, unidirectional terraces separated by atomic steps. The green line represents the path for the height profile provided in Figure 1C, which supports the average step height of ~7 nm and terrace width of ~85 nm, consistent with the nominal miscut angle of 5° (calculated to be 4.7°). Minor local deviations in step width are typical of thermally annealed vicinal surfaces [21], but none disrupt the overall directed terrace structure needed to promote macroscopic anisotropy. Figure 1B shows a higher magnification image confirming flat terrace surfaces and low defect density, important for epitaxially grown NiO. Measured terrace width (~85 nm) and step height (~7 nm) were consistent with the nominal 5° miscut and cross the known range of width and step height for influencing resistive switching and electrocatalytic activity in oxide thin films [32,33]. Although atomic-scale defect profiling (e.g., via TEM or PAS) was not performed, the strong correlation between morphologic and deterministic device outcomes (Section 3) supports the observation that step-guided vacancy migration—not random defect distribution—governs anisotropic transport.

The stepped morphology of the vicinal MgO(100) substrate is key to the growth mode of NiO epitaxially. NiO grows coherently in a rock-salt structure with a low lattice mismatch (0.86%) with MgO. The 5° miscut provides unidirectional terraces separated by atomic steps in the ⟨011⟩ direction in a manner that promotes step-flow growth. In the step-flow growth, adatoms incorporate at step edges preferentially because they have higher binding energies and shorter diffusion environments. The AFM suggests that the NiO film maintains the underlying terrace morphology, which characterizes the layer-by-layer growth due to the absence of island formation (Figure 1B). The anisotropic surface energy—lower parallel to less dangling bonds—drives the orientation relationship across the terraces and contributes to the formation of atomically persistent, smooth, continuous films that retain the macroscopic anisotropic character of the mesa. This growth mode directed by the terrace morphology creates anisotropic distributions of defects and paths for strain relaxation that may provide the basis for the observed directional resistive switching. These crystallographic and kinetic factors contribute to the electrical anisotropy we have observed.

To quantify the strain induced due to the substrate and its probable effects on switching, we derived the vicinal miscut angle and the lattice mismatch from the possible measurements of the film lattice parameters. The miscut angle was calculated from terrace width (W ≈ 85 nm) and step height (h ≈ 7 nm) was measured with the AFM:

(1) $$\theta =arctan\left(\frac{h}{W}\right)=arctan\left(\frac{7}{85}\right)\approx 4.7{}^{\circ}$$

in agreement with the nominal 5° vicinal. The lattice mismatch (in-plane) between the substrate and film, defined as $\varepsilon_{\left|\right|}^{mis}$ = $\frac{\left(a_{sub}-a_{0}\right)}{a_{0}}$ using $a_{sub}$(MgO) = 4.212 Å and a_0_ (NiO) = 4.176 Å, gives

(2) $$\varepsilon_{\left|\right|}^{mis}=\left(\frac{4.212-4.176}{4.176}\right)\approx 8.6\times 10^{-3}\;(\approx 0.86\%\;\mathrm{tensile}),$$

so, a fully coherent NiO film would be tensile in-plane. In practice, XRD reciprocal-space maps (RSMs) are used to measure the film in-plane and out-of-plane lattice parameters: from a measured 2θ for an (hkl) reflection, one computes the spacing d = λ/(2sin θ), and for a cubic film = $a_{\left|\right|}\sqrt{h^{2}+k^{2}+l^{2}}$.

(3) $$\varepsilon_{\left|\right|}^{mis}=\left(\frac{\left(a_{\left|\right|}-a_{0}\right)}{a_{0}}\right),\;\;\varepsilon_{⊥}\left(\frac{\left(a_{⊥}-a_{0}\right)}{a_{0}}\right)$$

where $a_{\left|\right|}$ and $a_{⊥}$ are the in-plane and out-of-plane lattice constants extracted from RSM. For a biaxially strained cubic film, the elastic coupling gives a relation between $\varepsilon_{⊥}$ and $\varepsilon_{\left|\right|}$:

(4) $$\varepsilon_{⊥}=\frac{2v}{1-v}\varepsilon_{\left|\right|}$$

with NiO’s Poisson ratio *ν* ≈ 0.21–0.25 ν ≈ 0.21–0.25 (reported for single-crystal NiO [33,34]; the expected out-of-plane strain is *ε* ⊥ ≈ −0.56% ε ⊥ ≈ −0.56% to −0.72% for a fully coherent film. In 30 nm films, partial relaxation at step edges is anticipated, which can be directly probed by RSM or TEM-GPA strain mapping. Such step-induced strain gradients are known to alter oxygen vacancy energetics and diffusion barriers in NiO [35,36], leading to spatial variations in filament nucleation and consequently systematic differences in forming and set/reset voltages. In NiO, however, nickel vacancies—not oxygen vacancies—are the primary charge carriers and filament constituents under p-type conditions; tensile strain is known to reduce nickel vacancy formation energy and enhance their mobility, thereby facilitating controlled filament growth [35,37]. We therefore quantify the miscut (4.7°), mismatch (+0.86%), and extract as *a* ∥, *a* ⊥ a ∥, a ⊥ from RSM to compute *ε* ∥, *ε* ⊥ ε ∥, ε ⊥, correlating these values with local I–V and c-AFM switching behavior.

**Figure 2:**
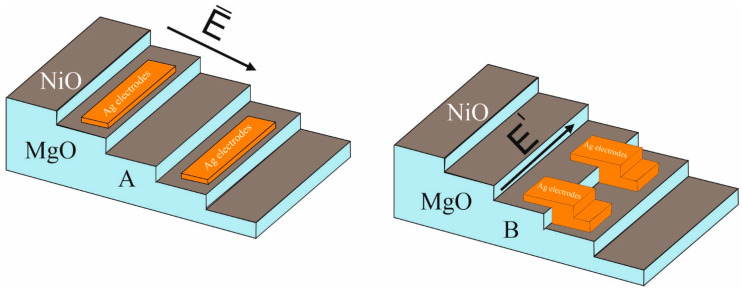
3D illustrations of Ag electrodes on NiO thin films: (**A**) parallel configuration; (**B**) perpendicular configuration. The arrow indicates the direction of the applied electric field (E).

Figure 2 shows 3D diagrams of Ag electrodes on NiO thin films grown on stepped MgO substrates. In configuration Figure 2A, the Ag electrodes are aligned parallel to the MgO substrate steps, promoting a uniform electric field distribution along the step edges. In configuration Figure 2B, the electrodes are arranged perpendicular to the substrate steps, which may cause non-uniform electric fields and localized stress, potentially resulting in higher forming voltages and less reliable switching behavior.

## 3. Results and Discussions

The resistance of the NiO thin film that was deposited at room temperature was above 200 MΩ. To lower this resistance and improve the quality of the film, the sample was post-annealed in a furnace for 1 h at 400 °C in ambient air. The post-annealed resistance measurements taken using the probe station showed a rapid decrease in resistance to 25 MΩ. This reduction was due to the increased concentration of nickel vacancies from exposure to an oxygen atmosphere during the anneal. Nickel vacancies behave as acceptors and generate holes as majority carriers under oxidizing conditions, which increases the p-type conductivity in NiO. Nickel vacancies under oxidizing conditions are formed at equilibrium under the Kröger–Vink notation:

(5) $$2{Ni}_{Ni}+\frac{1}{2}C_{2(g)}\to {2V}_{Ni}^{\prime \prime \prime }+2NiO+3h^{.}$$

In air at 400 °C, the elevated oxygen partial pressure shifts the balance to the right, favoring the production of $V_{Ni}^{\prime \prime \prime }$ and holes (h^.^) that act as majority carriers in p-type NiO. The temperature is high enough to enable Ni^2+ i^ on mobility (vacancy equilibration), but not high enough to induce Mg diffusion from the substrate or NiO decomposition [37,38]. One hour of annealing permits the system to reach a thermodynamic equilibrium, verified by the consistent post-annealing resistance of ~25 MΩ (across multiple samples). Such controlled defect engineering is critical to reproducible resistive switching. The coherent tensile strain (+0.86%) imposed by the MgO substrate aids nickel vacancy formation, demonstrated by the post-annealing resistance changing from 200 MΩ to 25 MΩ. This strain-mediated defect engineering reduces the energy barrier to filament nucleation, allowing soft electroforming in the 20–70 V range when aligned with the electric field direction. However, strain in the perpendicular configuration is not uniform along y-direction. Sharp step edges create localized tensile/compressive gradients that increase the electric field resulting in dielectric breakdown while switching. Substrate-induced strain therefore works in concert with step orientation to determine switching reliability. Nickel vacancies enhance p-type conductivity, improving the film’s semiconducting properties and reducing resistance.

**Figure 3:**
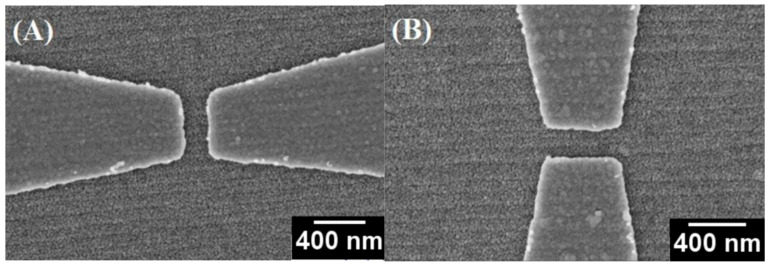
SEM images of Ag electrodes on NiO thin films. (**A**) Electrodes aligned parallel to the electric field. (**B**) Electrodes aligned perpendicular to the electric field.

SEM images of silver (Ag) electrodes deposited on the NiO thin films are shown in Figure 3. The electrodes were aligned to be either parallel (2A) or perpendicular (2B) to the stepped substrate topology. The electrodes were lithographically patterned to be 500 nm wide with an inter-electrode gap of 180 nm.

**Figure 6:**
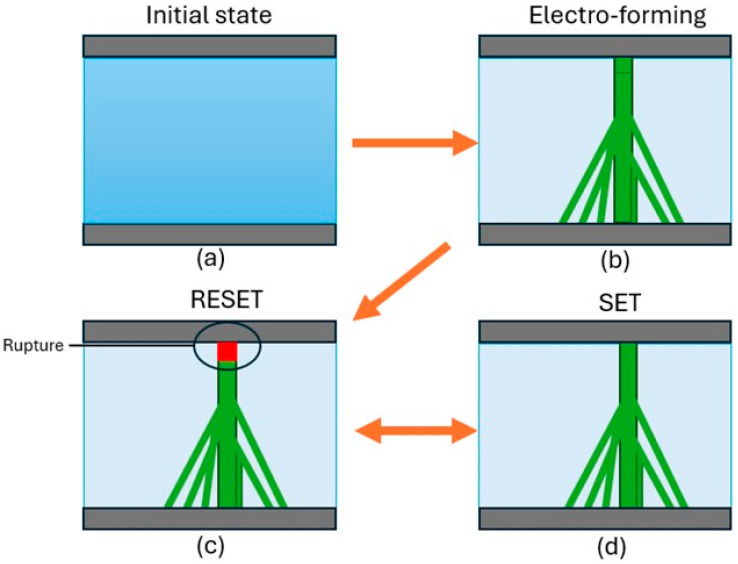
Schematic representation of conductive filament behavior under parallel and perpendicular electric fields. (**a**) Initial state; (**b**) electroforming of filament; (**c**) break (RESET); (**d**) reversible SET. For the parallel configuration, unipolar switching occurs via controlled nickel vacancy migration along terraces and allows for gradual, reversible filament growth, while requiring somewhat lower forming voltages. For the perpendicular configuration, increased electric fields at step edges promote dielectric breakdown and catastrophic failure, with all resulting in larger threshold voltages and irreversible behavior. Reproduced from Bulja et al. [38].

Electrical measurements were carried out using a two-probe system with a Keithley 2400 source meter (Keithley Instruments, Inc., Cleveland, OH, USA). Probe station tungsten probes were used to connect to the 100 × 100 μm electrode pads on the sample. The I–V properties of the devices were measured using voltage sweeps under DC voltage excitation at room temperature. To avoid permanent damage to the device while performing the forming voltage step, the compliance current was limited to 2 μA. In Figure 6, we provide additional evidence of local damage and pathways for breakdown by showing filament dynamics with different field orientations.

### 3.1. Electric Field Applied Perpendicular to the Steps

**Figure 4:**
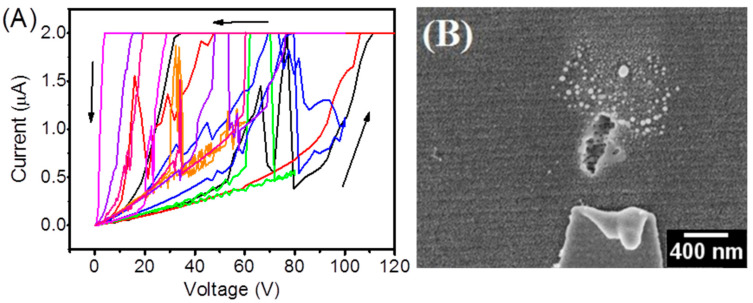
(**A**) Voltage sweep attempts during electroforming for eight devices with electric field applied perpendicular to steps. Each color represents a different device; arrows indicate sweep direction (upward and downward). All devices failed before achieving a stable LRS. (**B**) SEM image showing catastrophic breakdown (~100 nm holes) at step crossings.

In the first round of experiments, a substantial electric field was applied, normal to substrate steps, to activate the resistive switching mechanism. Figure 4A shows that all eight devices with the electric field applied perpendicular to the steps were found to fail during electroforming attempts, so no stable low-resistance state could be established. The magnitude of 120 V reported is the practical limit of our testing voltage range before this dielectric failure manifested itself. In this configuration, a value of forming voltage was not recorded at all. The SEM images (Figure 4B) associated with the damaged devices highlight severe local damage, including large holes (~100 nm) related to the electrodes, and all devices failed to operate after voltage was applied to the form. While the study did not conduct compositional analysis (i.e., EDS or XPS), the localized nature of the damage (i.e., damage only occurring in perpendicular devices and under the identical application of electric field) strongly suggests that the degradation was primarily due to the bolt of electric field enhancement at the step edges and did not stem from the interdiffusion of materials or chemical degradation of materials. Future studies will include chemical mapping to look for the potential of Ag diffusion (or oxygen vacancy accumulation) at the site of breakdown. This result is consistent with the fact that perpendicular fields in step edges produce strong local enhancement leading to catastrophic dielectric breakdown, while parallel fields reduce local stress and support filamentation driven by oxygen vacancies, aided by a lowered forming voltage, thereby defining substrate-induced thickness variations as the primary distinction between failure and stable resistive switching.

An electric field applied perpendicular to substrate steps produces strong local field enhancements due to sharp edges and corners that exceed the breakdown strength of NiO. This results in catastrophic dielectric breakdown with an unstable filament. Evidence from the SEM shows damage to the electrode and film from this dielectric breakdown process. However, when the field is applied parallel to the steps, local enhancement and voltage drop are reduced, as the forming voltage is lower, which allows for gradual soft breakdown, resulting in the stable formation of filaments as a result of the movement of the nickel vacancy particles. A difference in the breakdown mechanisms occurs due to variation in thickness based on substrate orientation. When a breakdown occurs in the perpendicular direction, it happens at an edge (localized failure). In the parallel orientation, the voltage drops, and gradual breakdown occurs uniformly throughout the film (with stable resistive switching). Moreover, filament growth is influenced by additional microstructural parameters even in a parallel electric field. Grain size and uniform vacancy distribution also affect filament growth via oxygen vacancy migration and thus influence filament nucleation and growth.

### 3.2. Electric Field Applied Parallel to the Steps

**Figure 5:**
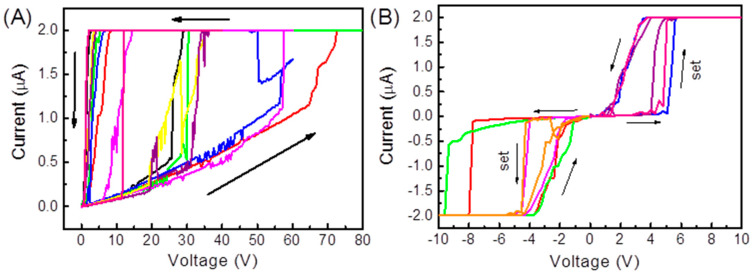
(**A**) Forming voltage applied parallel to steps. Each color represents a separate device; arrows indicate sweep direction. (**B**) Set voltage measurements indicating unipolar switching. Arrows denote switching direction; “set” indicates transition from high-resistance state (HRS) to low-resistance state (LRS).

Following this, inducing the electric field along the direction of the substrate steps was tested to assess anisotropic effects. With the same compliance current for forming (2 μA), the voltage range for forming was 20 V to 70 V, showing the inherent stochastic nature of filament nucleation in these oxide-based ReRAM devices [37,38]. Notably, all eight devices with parallel electrodes (yield 100%) formed and could reliably switch unipolar, while none of the perpendicular-led devices survived (Section 3.1). This deterministic pattern—100% functional yield and consistent HRS/LRS ratio (~10^2^) across all devices—cannot be more different than the large resistance variations and stochastic forming found in standard NiO ReRAM devices (Table 1). This physicochemical architecture, in tandem with morphology-directed filament nucleation, uncovers a major barrier for array scalability for NiO ReRAM. This is a dramatic difference, as all devices are successful or all devices fail solely based upon the orientation of the field. The stepped substrate provides functionally uniform anisotropy. The relevant feature is not just localized effects; it is the macroscopic arrangement of the steps that draws vacuoles and limits localized breakdown from occurring while the electric field runs parallel to the terraces. Figure 5A shows the forming voltage for the devices parallel to the elongated electric field vectors. Unlike the perpendicular outcome, all eight devices could survive the forming process.

The device’s original resistance was around 30 MΩ. Once conducting filaments were formed, resistance lowered to 1 MΩ. In Figure 5B we can see the set voltage as determined by the switching cycles (±5 V) shows unipolar switching behavior. In this fashion, both the set (low-resistance state) and reset (high-resistance state) happen at the same voltage polarity. The HRS/LRS ratio was approximated around 102 when using ±5 V. Importantly, the difference between binary outcomes, wherein 100% functional yield is achieved in parallel and 0% in perpendicular showed that substrate-induced anisotropy can eliminate catastrophic failures and rescale device reproducibility, which is necessary for ReRAM’s scalable device integration. The difference in behavior between the parallel and perpendicular devices relies on the steps that accomplish two tasks: (1) modifying local electric field distribution, and (2) providing pathways for nickel vacancy directional movement. The schematic in Figure 6 illustrates how parallel alignment enables directional filament formation while orientation perpendicular to the steps facilitates localized failure. When the field is applied perpendicular to the steps, the ~7 nm height discontinuity at the step edges acts as a topographic singularity that creates high electric field conduction like that observed at lightning rods, locally exceeding the dielectric breakdown strength of NiO (~1–2 MV/cm). This state can be evidenced by SEM (Figure 4B), which shows catastrophic failures with ~100 nm holes exactly at step crossings. Conversely, when the field is parallel to steps, the smooth terrace morphology provides a uniform electric field distribution due to the presence of overlying steps acting as defect migration pathways for nickel vacancies, as anticipated from lower migration barriers along the ⟨011⟩ direction resulting from surface energy anisotropy in vicinal oxides [21,23,25]. The bimodal behavior—failures at perpendicular translational orientations but success at parallel translational orientations—indicates that steps serve dual purposes, which are (1) to modulate the electric field, and then (2) to be defect migration pathways. This is why all parallel devices (8/8) were able to reproducibly fabricate filaments in the voltage range (20–70 V), and all perpendicular devices (0/8) failed irreversibly under the same conditions. The condition of being bimodal (success vs. failure) is not coincidental or random, but deterministic in that the type of steps in relation to the applied electric field direction is responsible for whether pathway filaments are nucleated. Even without directly imaging defects, the reproducibility of switching across all parallel devices demonstrates that the morphology of the substrate itself serves as a stand-in for defects’ ordering. In NiO, nickel vacancies are the primary mobile species under an oxidizing anneal [37,38], and vacancy migration is known to be sensitive to strain and to surface topology [35,36]. In this way, nickel vacancies play a twofold role: they regulate the overall p-type conductivity of the film and assume the role of the mobile ionic species that form conductive filaments during the electroforming process. The anisotropic behavior, therefore, suggests a morphology-templated defect landscape where the width of terraces and the height of steps become the spatial scale for filament guidance.

The structure and stoichiometry of NiO films are also recognized to influence defect distribution and filament formation, and techniques like XRD or XPS would help elucidate. Previous work concluded that resistive switching in NiO was stable over film structures, both polycrystalline [37] and amorphous [38], implying that switching in this case is not dependent on crystallinity. Our data also convey the influence of substrate steps, with devices with electric field applied parallel to steps being more reproducible, and requiring lower forming voltage than the perpendicular case, where the step edge can cause premature breakdown by sharpening the field. The three fundamental criteria required for this anisotropic behavior are (i) structural compatibility (rock-salt MgO and NiO have <1% lattice mismatch, allowing for coherent growth), (ii) defect-mediated switching driven by mobile cation vacancies (e.g., the Ni vacancy in NiO, akin to Fe vacancies in Fe_3_O_4_), and (iii) unidirectional step morphology to break the in-plane symmetry. These criteria are satisfied by a wide class of transition metal oxides, including but not limited to CoO, MnO, and doped SrTiO_3_, suggesting that step-engineered substrates provide a general strategy of spatially guiding filament formation to increase device yield.

To place our results in context, we present a comparison of important performance parameters for NiO-based ReRAM devices that have been reported in the literature in Table 1. Previous studies have demonstrated moderate HRS/LRS ratios, but experience high forming voltages, stochastic yield, and no control of filament location. In contrast, our strategy utilizes substrate-induced anisotropy to achieve deterministic switching—100% of parallel-aligned devices formed reliably at ≤70 V while perpendicular devices uniformly failed. Using this binary outcome, we conclude that morphological templating, not just material selection, can address the reproducibility dilemma for ReRAM.

## 4. Conclusions

In addition to showing anisotropic resistive switching in NiO, this work establishes design principles for engineering defect migration pathways in a variety of oxide electronic materials. By connecting substrate step-induced strain, electric field distribution, and vacancy migration, we show that the substrate morphology is not just a template for growth, but an active control parameter for resistive switching reliability, and similar principles would apply even to other oxide systems that are grown epitaxially. The reproducible anisotropic switching, despite the residual local non-uniformity associated with small deviations in the step size or spacing, suggests that macroscopic step alignment can provide sufficient control to dictate filament formation in NiO-based devices, rather than perfect atomic alignment. Devices where the electric field was applied along the substrate step direction demonstrated lower forming voltages (20–70 V), and increased device reliability and yield (100% failure free), unipolar switching (HRS/LRS ≈ 10^2^ at ±5 V), and levels of switching behavior, while devices with the electric field applied perpendicular were destroyed (0/8 working). This complete success vs. total failure demonstrates that the strain and step density associated with the substrate are critical considerations in the synthesis of thin films or other bad oxides.

These findings show that the directionality of substrate steps can be used to modify the resistive switching behavior of NiO. In this research, high-temperature annealed MgO was used to decouple the essential mechanisms; however, it is important to state that this step-guided filament control scheme is transferrable to CMOS-compatible platforms by using nanoscale templates (e.g., easily patterned SiO_2_ and Al_2_O_3_) through low-temperature (<400 °C) processing for future integration with Si ICs, but still permits anisotropic switching and low variability. Similarly, the anisotropic charge transport pathway would be beneficial to improve the electrocatalytic and photovoltaic performance of NiO-based energy devices, e.g., improving OER efficiency directly from directional (hole) conduction. Specifically, the step-guided terrace morphology increases the density of catalytically active step edge sites, and controlled nickel vacancy concentration increases hole conductivity—both are critical to efficient OER. Optimized directional hole transport along ⟨011⟩ terraces reduce bulk recombination and promotes surface oxidation kinetics which suggests that the substrate-engineering method applied to ReRAM can lead to optimized NiO-based electrocatalysts.

Future studies will investigate how resistive switching and spin-dependent phenomena will vary with different step densities and miscut angles, further contributing to device performance refinement. Additionally, in situ c-AFM mapping and chemical characterization (XPS/EDS) of breakdown sites will be performed to directly visualize filament paths and eliminate interfacial reactions as a principal failure mechanism.

## Figures and Tables

**Table 1:** Comparison of NiO-based ReRAM performance metrics across recent studies.

Study	Substrate	Electrode	HRS/LRS	Forming Voltage	Device Yield	Key Limitation	Anisotropy Control
Ahn et al. [37]	Si/SiO_2_	Pt/NiO/Pt	~10^1^	~100 V	Not reported	High variability	No
Bulja et al. [38]	Glass	Ag/NiO/Ag	~10^2^	30–80 V	Moderate	Stochastic forming	No
Shin et al. [35]	SrTiO_3_	Pt/NiO/Pt	~10^3^	>100 V	Low	High voltage, poor yield	No
This work	5° miscut MgO	Ag/NiO/Ag	~10^2^	20–70 V	100% (parallel)	Requires epitaxial substrate	Yes (step-guided)

## Data Availability

Data will be available at reasonable requests to the authors.
